# Vulture distribution and people perception of vultures in Pokhara Valley, Nepal

**DOI:** 10.1002/ece3.8528

**Published:** 2022-01-12

**Authors:** Hemanta Dhakal, Hari Prasad Sharma, Christopher J. W. McClure, Munir Virani, Brian W. Rolek, Narendra Man Babu Pradhan, Krishna Prasad Bhusal

**Affiliations:** ^1^ Department of Zoology, Prithvi Narayan Multiple Campus Pokhara Tribhuvan University Pokhara Nepal; ^2^ Central Department of Zoology Tribhuvan University Kathmandu Nepal; ^3^ The Peregrine Fund Boise Idaho USA; ^4^ Bird Conservation Nepal Kathmandu Nepal; ^5^ International Union for Conservation of Nature Kathmandu Nepal

**Keywords:** abundance, birds, conservation, habitat loss, population, vulture

## Abstract

Due to an abundance and diversity of vultures, Nepal is one of the most important countries for vulture conservation. Within Nepal, the Pokhara Valley is especially significant. We examine the distribution of vultures within the Pokhara Valley by conducting counts at 11 potential feeding or roosting sites using point count method. We further surveyed people of the valley regarding their perception of vulture ecology and conservation, knowledge of diclofenac use within the valley, and burial of livestock carcasses. We detected eight species of vultures, four of which are currently threatened with extinction. White‐rumped vulture *Gyps bengalensis*, Egyptian vulture *Nephron percnopterus*, and Himalayan vulture *G*. *himalayensis* were the most abundant. Almost all respondents (98%) had sighted the vultures in the wild. Formally educated respondents reported seeing vultures’ slightly more than nonformally educated respondents. Fifty‐eight percent respondents suspected habitat loss was the major threat for the vulture population decline in Pokhara Valley, and 97% respondents were not aware of any diclofenac use. The knowledge of vultures in people with different age groups suggests a more awareness programs are needed for local people, especially those who carry out animal husbandry and provide livestock to the vulture restaurant.

## Graphical abstract

Among the eight species of vultures, four of which are currently threatened with extinction in our study sites. White‐rumped Vulture, Egyptian Vulture, and Himalayan Vulture were the most abundant species. Sites with food resources including a landfill and vulture restaurant had the highest vulture abundance. Almost all respondents were known about vultures and 98% of respondents reported seeing vultures in the wild.

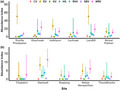

## 1 INTRODUCTION

Old World vultures comprise the most threatened group of birds in the world (Buechley & Şekercioğlu, 2016a, 2016b), with 80% of species experiencing global population declines and 75% listed as either endangered or critically endangered by the International Union for the Conservation of Nature (IUCN; McClure et al., 2018). The greatest threats to these scavengers are various forms of poisoning such as using anti‐inflammatory veterinary drug Diclofenac on livestock treatment (Buechley & Şekercioğlu, 2016a; Margalida & Ogada, 2018; McClure et al., 2018; Ogada et al., 2012). During what is now known as the “Asian Vulture Crisis,” vulture populations across South Asia were decimated by veterinary use of nonsteroidal anti‐inflammatory drugs (NSAIDs; Margalida & Ogada, 2018; Ogada et al., 2012; Pain et al., 2008). In particular, diclofenac caused catastrophic declines of >95% in three species of *Gyps* vultures after being introduced in the 1990s (Oaks et al., 2004; Prakash, 1999; Prakash et al., 2003, 2012).

In response to these population declines, the governments of India, Pakistan, and Nepal banned the importation, production, and sale of veterinary diclofenac in 2006, with Bangladesh following in 2010 (Margalida & Ogada, 2018; Pain et al., 2008). Conservationists also promoted nontoxic meloxicam as an alternative to diclofenac (Swan et al., 2006; Swarup et al., 2007) and established Vulture Safe feeding sites (Vulture Restaurants) where diclofenac‐free carcasses are provisioned as food for vultures (Chaudhary et al., 2012). Importantly, established Vulture Safe Zones also requires working with local communities to remove stocks of diclofenac, educate potential diclofenac users about its deleterious effects, and monitor the potential drug users in surrounding areas (Chaudhary et al., 2010, 2012). Following these conservation actions, some populations of Gyps vultures in South Asia seem to have stabilized from 2012 onwards (Chaudhry et al., 2012; Galligan et al., 2019; Prakash et al., 2012).

Despite some encouraging results, the threat of NSAIDs remains (Cuthbert, Dave, et al., 2011; Cuthbert, Taggart, et al., 2011; Margalida & Ogada, 2018). Illegal use of diclofenac in veterinary medicine still occurs to varying degrees across Africa and South Asia (Botha et al., 2017; Cuthbert, Dave, et al., 2011; Cuthbert, Taggart, et al., 2011), and several legal NSAIDs are toxic to vultures (Cuthbert et al., 2007, 2016; Naidoo et al., 2010). Less than one percent of livestock carcasses need to be contaminated with diclofenac to cause catastrophic losses observed during the Asian Vulture Crisis (Green et al., 2004). Actions including establishing Vulture Safe Zones and educating local people of the dangers of diclofenac therefore remain conservation priorities. In fact, according to IUCN Red List assessments, “education and awareness” and “law and policy” are the two most pressing conservation needs for Old World vultures (McClure et al., 2018)—highlighting that both legislative and educational actions are needed to assuage the threat of poisoning (Parvanov et al., 2018).

**FIGURE 1:**
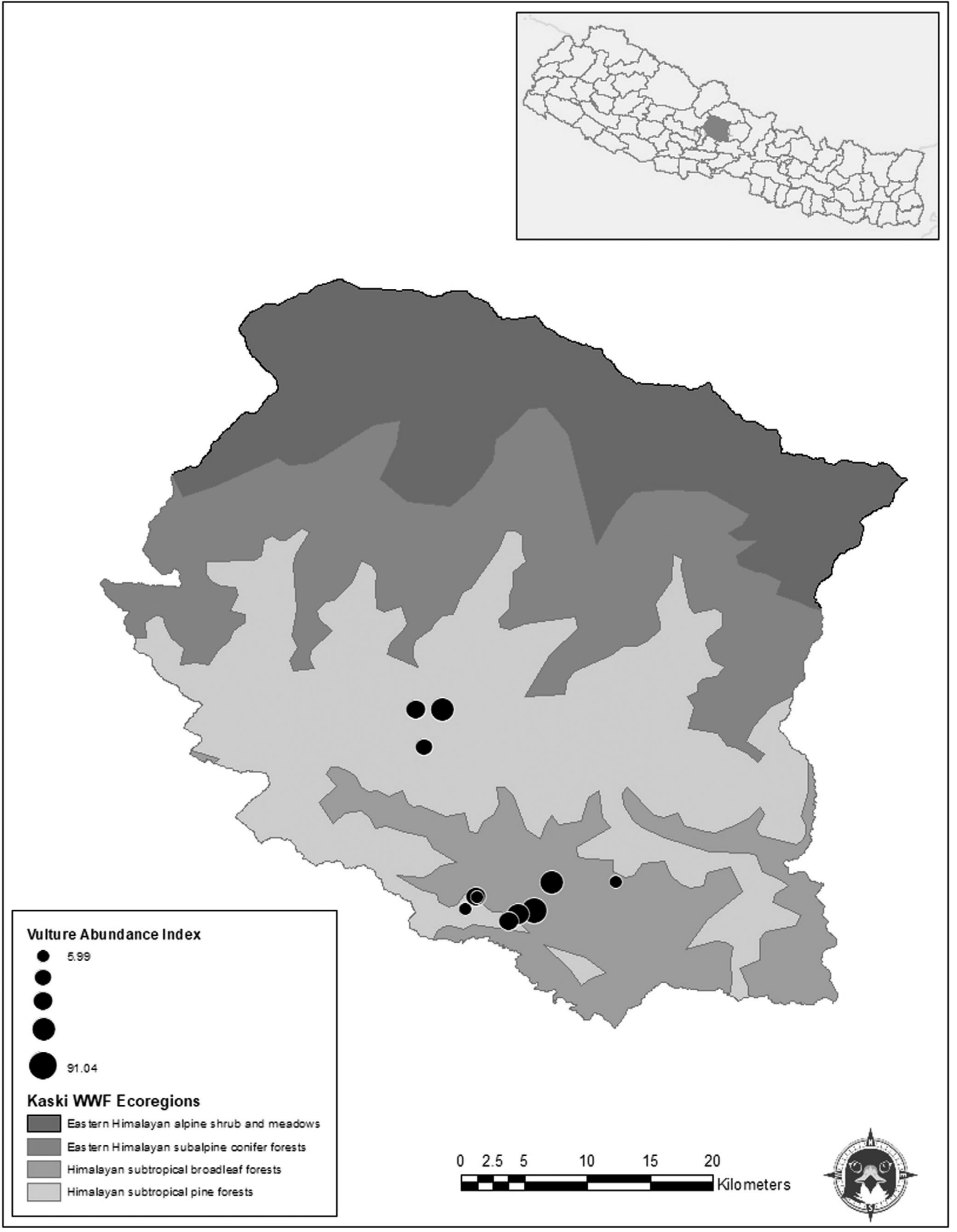
Map of the Kaski District and the location of the Kaski District within Nepal (inset). Circles indicate vulture survey locations and are scaled by their overall relative abundance. Gray shading indicates World Wildlife Fund Ecoregions (Olson et al., 2001)

Among nine species recorded in Nepal, Himalayan Vulture *Gyps himalayensis*, Slender‐billed vulture *G*. *tenuirostris*, White‐rumped vulture *G*. *bengalensis*, Red‐headed vulture *Sarcogyps calvus*, Egyptian vulture *Nephron percnopterus*, and Bearded vulture *Gypaetus barbatus* breed in Nepal, Long‐billed vulture *G*. *indicus* is vagrant and other two species Eurassion Griffon *G*. *fulvus*, and Cinereous vulture *Aegypius monachus* are winter visitors (Department of National Parks and Wildlife Conservation: Bhusal et al., 2019; Dhakal et al., 2019; DNPWC, 2015). Nepal is one of the most important areas on Earth for raptor conservation, especially in the context of vulture declines (Prakash et al., 2012). This relatively small nation contains six of the 10 most critical Important Bird and Biodiversity Areas (IBAs) for raptors and contains the 10th greatest number of declining raptor species of all nations (42 species; McClure et al., 2018). Indeed, the concept of Vulture Safe Zones was first successfully implemented in Nepal (Chaudhary et al., 2010). The Kaski District of Nepal might be particularly important—containing five ecoregions (Figure 1; Olson et al., 2001) and the Annapurna Conservation Area, which is the sixth most important IBA for raptors, globally (McClure et al., 2018). This district has one Vulture Safe Feeding Site at Ghachowk. Because of such diversity of vultures with this small area, it is important that conservationists understand the local people’s perception toward vultures.

Here, we report the results of questionnaires completed by residents of the Kaski District of Nepal regarding their knowledge of vulture biology and ecosystem services, nearby use of diclofenac, and recent population status. We further report counts of eight vulture species, of which four are currently threatened with extinction (BirdLife International, 2019; Dhakal et al., 2019; Inskipp et al., 2016). We specifically emphasize local knowledge of the vulture (see Table 1 for scientific names) because these are rare (Chaudhary et al., 2012), critically endangered (BirdLife International, 2019), and the least‐studied vulture species (Buechley et al., 2019) for their long‐term conservation. The Pokhara Valley is one of the high human‐dominated area, which supports the occurrence of vulture species. People’s knowledge and perception on the importance of vulture in an ecosystem can help to develop policy for conservation of these species. However, we have little information on the vulture abundance, and people’s knowledge and perception in and around the residential areas. This information is pre‐requisite to update the existing conservation plan and to develop site‐specific management plan at one of the IBA in Nepal. Therefore, we aimed to provide site‐specific vulture abundance and people’s knowledge on vulture conservation near to their settlement.

**TABLE 1:** Common and scientific names, number of observations, and median, 2.5th, and 97.5th percentiles of relative abundances of vulture species across survey sites within the Kaski District, Nepal

Common name	Scientific name	Observations	2.5th	Median	97.5th
White‐rumped Vulture	*Gyps bengalensis*	814	7.01E−01	8.49	32.32
Egyptian Vulture	*Neophron percnopterus*	1,294	7.35E−01	6.23	74.96
Himalayan Vulture	*Gyps himalayensis*	619	2.03E−04	3.59	39.27
Red‐headed Vulture	*Sarcogyps calvus*	229	5.35E−05	2.04	10.55
Slender‐billed Vulture	*Gyps tenuirostris*	192	9.34E−06	0.74	9.29
Cinereous Vulture	*Aegypius monachus*	87	1.98E−05	0.23	5.28
Griffon Vulture	*Gyps fulvus*	90	2.80E−06	0.02	17.85
Bearded Vulture	*Gypaetus barbatus*	2	NA	NA	NA

Note

Note that abundance of Bearded Vulture was not statistically analyzed.

## 2 METHODS

### 2.1 Study area

We focused our study on the Pokhara Valley (28°7′N to 28°12′N, 84°5′E to 84°10′E) of Kaski District, Gandaki Province, Western Nepal (Figure 1). The district is situated at the center of Nepal, the elevation ranges from 450 m to 7969 m from the sea level. About 44.6% of the total area of the district is covered by forest (District Development Committee: DDC, 2005). The study area has a subtropical climate. The maximum and minimum temperatures were recorded 33°C in April and 5.6°C in January, respectively. The study area consists mainly of steep topography with many rocky cliffs. Vegetation is primarily Chir pine (*Pinus roxburghii)* forest in the high elevation areas along with Needle wood tree (*Schima wallichii*) and Indian chestnut (*Castanopsis indica*). At lower elevations, the predominant tree species are Sal (*Shorea robusta*) and Silk cotton (*Bombax ceiba*; Tamrakar 2008). The study area supports the occurrence of 462 bird species including nine vulture species. Among these bird species, 18 are globally threatened and 11 are near threatened (Ghimire et al., 2019). The Pokhara Valley is capital city of Gandaki Province, high human‐dominated area, and is well known for tourist area. Around 83% of people are literate (Central Buero Statistics: CBS, 2011).

### 2.2 Data collection

#### 2.2.1 Vulture

We performed fixed‐point surveys following Huff (2000) at 11 sites that we chose based on potential roosting and feeding habitat, and these sites cover the whole study area which comprises approximately 250 km^2^. The distance between first and last site was approximately 30 km apart and minimum distance between two sites was around 2 km. We recorded all individual vultures soaring, feeding, and roosting. We performed all surveys from vantage points that provided views of the entire survey sites. We surveyed four sites in a week for 3–4 h each time from September 2016 to August 2017. To avoid duplication of on vulture counting, 11 vantage points were divided into 4 sites and each sites had 2–3 vantage points. We recorded number of vultures at the same time covering each point by three observers, during each observation. All sites were covered in a month. We communicated each observer if the soaring vulture seems potentially overlapping between nearest sites to avoid the double count of individual. We recorded the number of each vulture species at every 30‐min interval, and we used the record of highest number of each vulture species from each station for data analysis. Surveys were conducted between 07h30 and 10h30 in the morning and 16h–18h in the evening during summer and 08h–11h30 in the morning and 15h–17h in the evening during winter.

#### 2.2.2 Questionnaire survey

Using a questionnaire (Appendix S1), we interviewed randomly selected respondents, who were living near to study area, that is, within 1 km periphery for questionnaire survey at each of the 11 vulture monitoring sites. We interviewed with 300 individuals, and the number of interviewee was varied according to sites (8–30 household) based on the household available within the 1 km periphery of study site. The interviewee was selected creating random number. The selected sample size for interview at each site was based on the 5% margin of the error at 95% confidence interval (Kreb, 2014). We surveyed only one adult (>16 years old) per household. We did not discriminate to the people based on education level, gender, ethnicity, or religion. We collected demographic data such as age, gender, and education. Furthermore, the questionnaire was designed to assess perception and knowledge of vultures as well as livestock holding practices including use of NSAIDS and methods of carcass disposal. In addition to the questionnaire, we showed pictures of different species of birds including all vulture species of Nepal, some eagle, and storks and asked them to identify which ones were vultures. The conduct of all aspects of this study was approved by the Department of National Parks and Wildlife Conservation, Nepal (permit DNPWC‐074/75‐2303).

### 2.3 Analysis

We implemented state‐space models in a Bayesian framework to estimate relative abundance of vulture species observed at each of our survey sites (Benson & McClure, 2019). A Bayesian framework allowed us to implement state‐space models while accounting for effects from survey effort on vulture counts. State‐space models can separate process from observation error in count data and can estimate imperfectly observed latent processes such as population fluctuations (Kéry & Schaub, 2012). These state‐space models accounted for imperfect detection and misclassification of species, but assumed an equal probability of these errors. Therefore, we estimated an improved yet imperfect index of relative abundance rather than counts.

We modeled monthly relative abundance (*N*) as a Markovian process: log (*N_t_*_+_ *_1_*_,_ *_s_*) = log (*N_t_*_,_ *_s_*) + *r_t_*_,_ *_s_* (Kéry & Schaub, 2012), where *r* is the stochastic change in vulture counts and can be interpreted as the monthly change in abundance over time (*t*) at each survey site (*Ss*). We included effort as a scaled and centered covariate (mean = 0 and SD = 1) to standardize counts to the mean time spent during surveys. *BpB* = 1*p* = 00 < *B* < 1*p* = 0*B* > 0*p* < 0*pp* = −0.001

We implemented models using JAGS (Plummer, 2003) and the package jagsUI (Kellner, 2016) in R (R Core Team, 2017) and used three chains with 300,000 iterations, burn‐in of 150,000, adaptation of 20,000, and we thinned one out of every 50 posterior draws resulting in three chains each having 3,000 posterior draws. We calculated the Gelman‐Rubin statistic ($\hat{R}$; Gelman & Rubin, 1992) for each parameter and determined convergence of chains when $\hat{R}$ < 1.1, and we created trace plots of parameter chains to assess convergence of chains. To further assess our models, we calculated the ratio of observation error to process error (i.e., the error ratio; Auger‐Méthé et al., 2016) and considered our models to be adequate when the probability that the error ratio >10 was >0.90. We used vague priors for all parameters because these priors produce similar estimates to those expected from a frequentist analysis of the same model where prior information does not influence estimates (Kéry & Schaub, 2012). We analyzed counts of all species except Bearded Vulture because it was only observed twice. A full description of the model and script for model implementation are archived at https://github.com/The‐Peregrine‐Fund/Nepal‐Vultures.

To compare species abundances, we calculated relative abundance across all months and study sites. To estimate an index of relative abundance that included all vulture species at a survey site, we summed each iteration of the posterior draws across all species. We present the median, 2.5th, and 97.5th percentiles for all reported estimates.

We examined the differences between education levels, age groups, and sexes regarding awareness of diclofenac, knowledge on the vulture species, and its ecosystem services. Our data were not normally distributed; therefore, we used Fisher’s exact test and a Kruskal–Wallis tests for binary and numeric questionnaire responses, respectively. We performed all analyses in R program (R Core Team, 2017).

## 3 RESULTS

**FIGURE 2:**
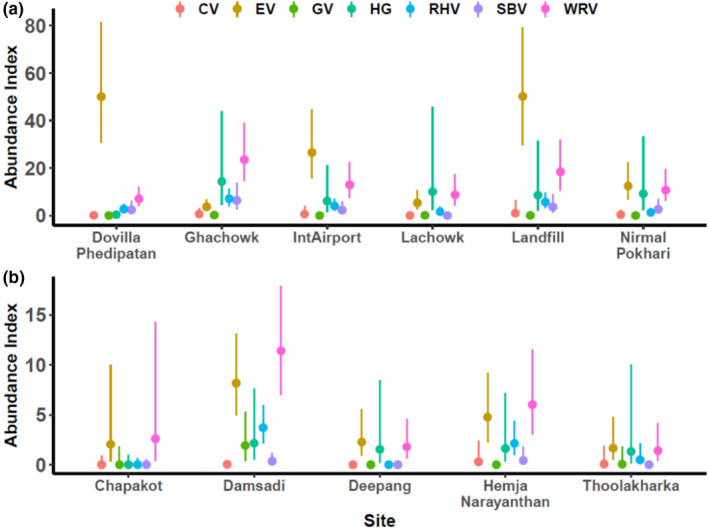
Relative abundance of vulture species per survey site within the Kaski District of Nepal. These relative abundances represent the median (dots) value of a given species during individual surveys. Thin lines represent the range between the 2.5th and 97.5th percentiles. Note that the axes in panels (a) and (b) are scaled differently such that sites with higher vulture abundances are in panel (a). CV = Cinareous Vulture, EV = Egyptian Vulture, GV = Griffon Vulture, HG = Himalayan Vulture, RHV = Red‐headed Vulture, SBV = Slender‐billed Vulture, and WRV = White‐rumped Vulture

We detected eight species of vultures, of which Egyptian vulture and Bearded vulture were detected the most and least often, respectively (Table 1). The species with the highest relative abundance per survey across sites were White‐rumped vulture (median = 8), Egyptian vulture (median = 6), and Himalayan vulture (median = 3) (Figure 2, Table 1, Table S1). Of the species analyzed, the three with the lowest relative abundance were Cinereous vulture, Griffon vulture, and Slender‐billed Vulture (Figure 2, Figure S1, Table S1). There was, however, substantial variance in relative abundance across sites for some species. For example, the Egyptian vulture was especially abundant at a few sites, leading to particularly skewed distributions of their survey‐wide relative abundance (Figure 2, Figure S1, Table 1). The three sites with the highest overall relative abundance were the Landfill, Dovilla, and Ghachowk, whereas the three sites with the lowest vulture abundance were Thoolakharka, Deepang, and Chapakot (Figure 2, Figure S1, Table S1). No species had *B* values with positive 2.5th percentiles (Table S2). The probability that the error ratio >10 was <0.90 for all species (Table S3).

**FIGURE 3:**
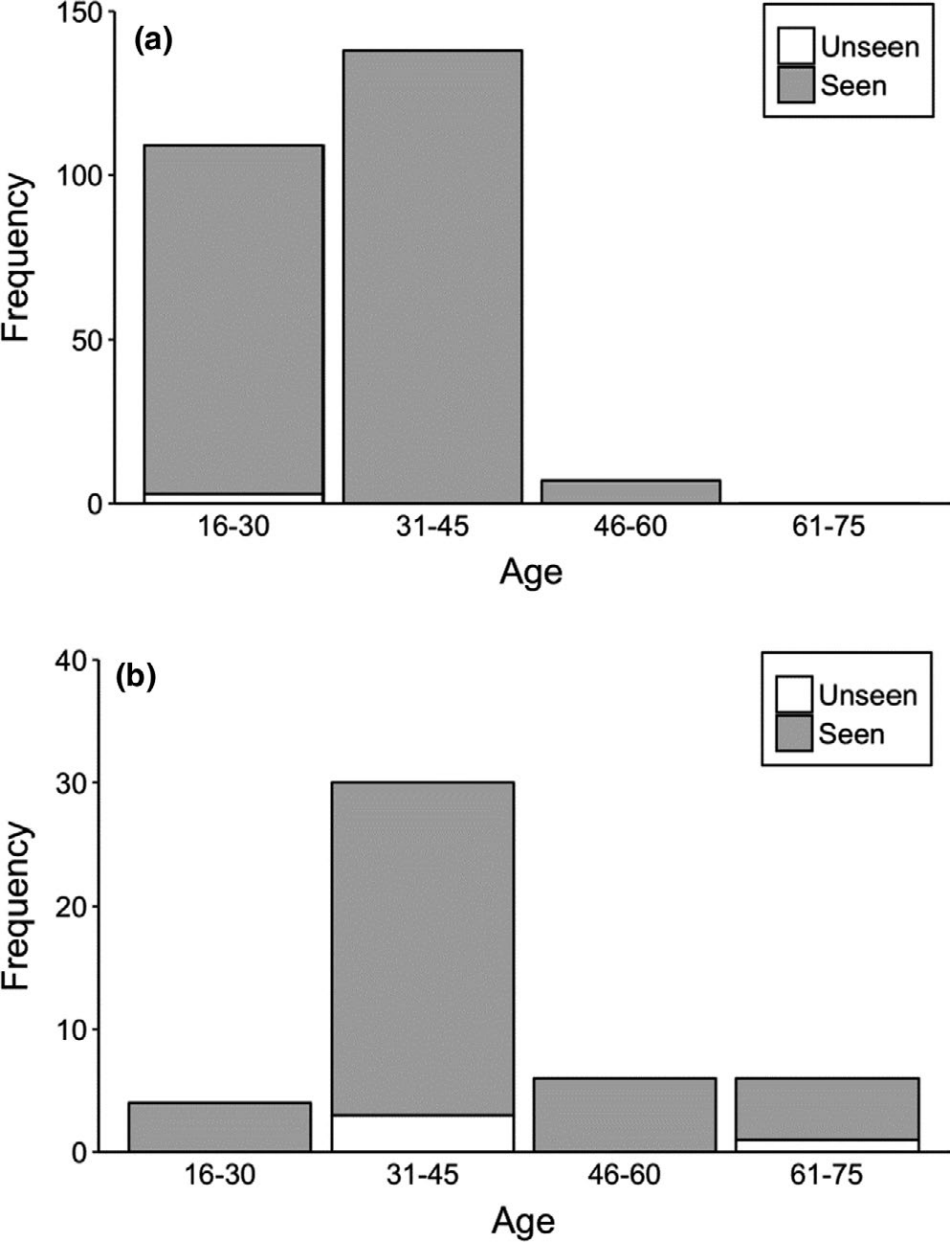
Differences in perceptions of respondents regarding whether they normally see vultures in the study area, (a) formally educated respondents and (b) non‐formally educated respondents

**FIGURE 4:**
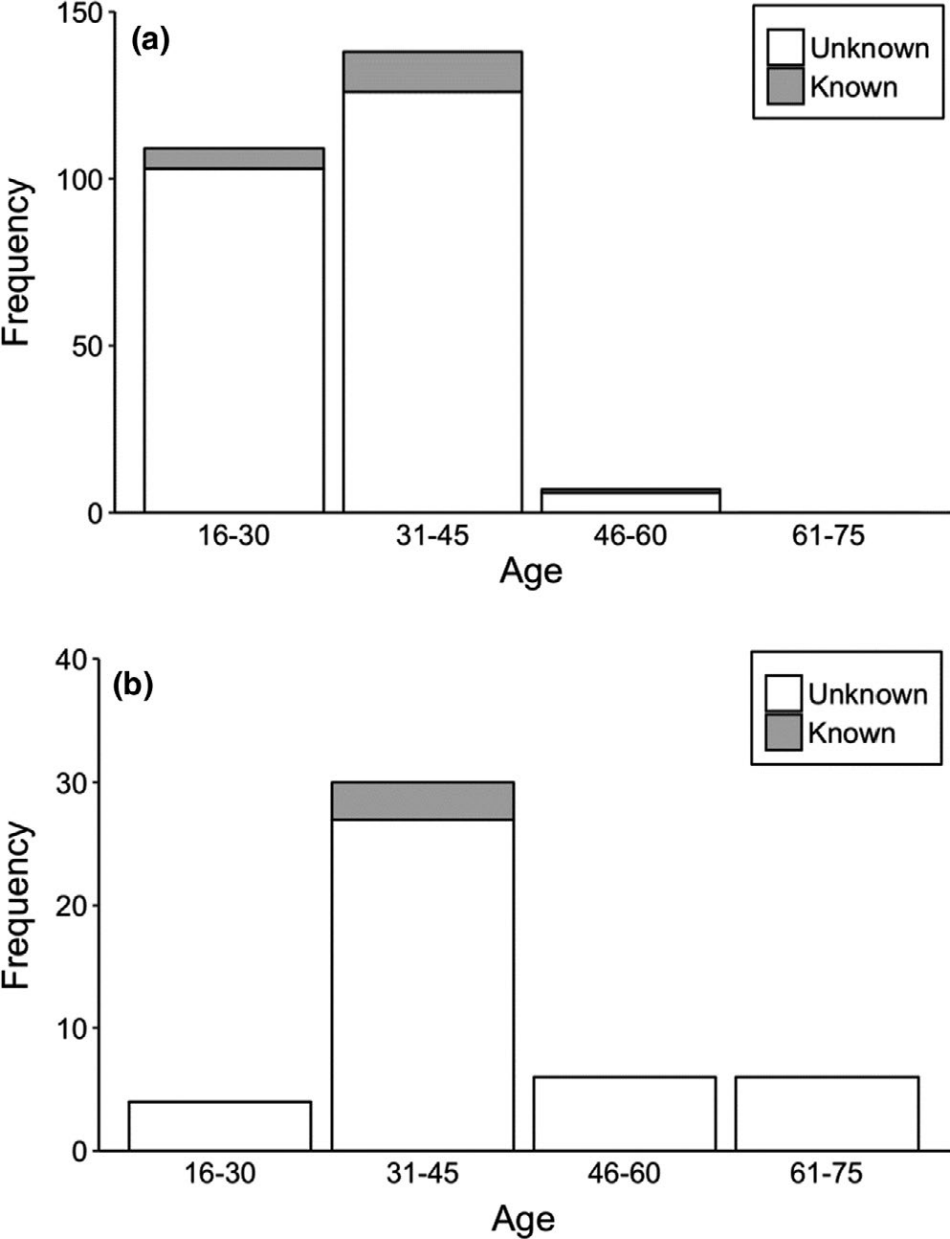
Differences in perceptions of respondents regarding where they normally see vultures’ nest in the study area, (a) formally educated respondents and (b) non‐formally educated respondents

**FIGURE 5:**
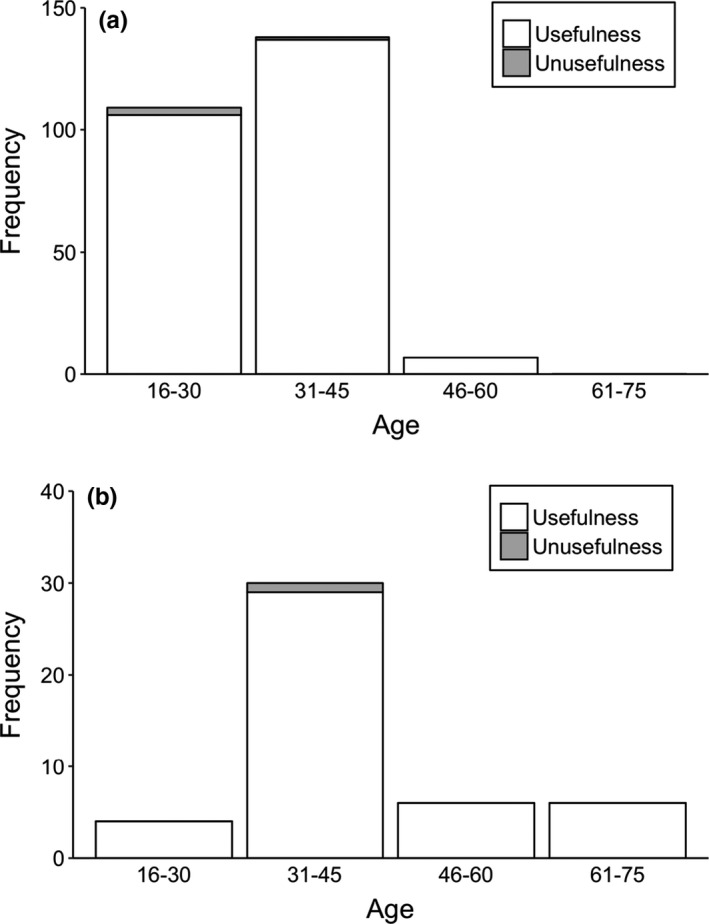
Differences in perceptions of respondents regarding the usefulness of vultures in the study area, (a) formally educated respondents and (b) non‐formally educated respondents

**FIGURE 6:**
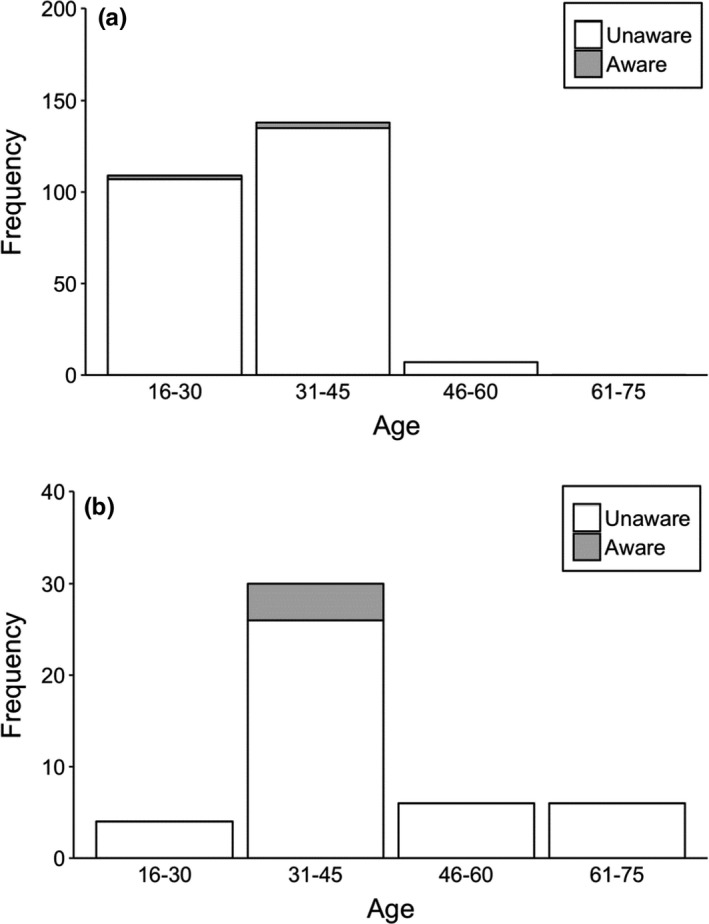
Awareness of respondents on diclofenac/other toxic chemical use in livestock and carcass with different age groups, (a) formally educated respondents and (b) non‐formally educated respondents

**FIGURE 7:**
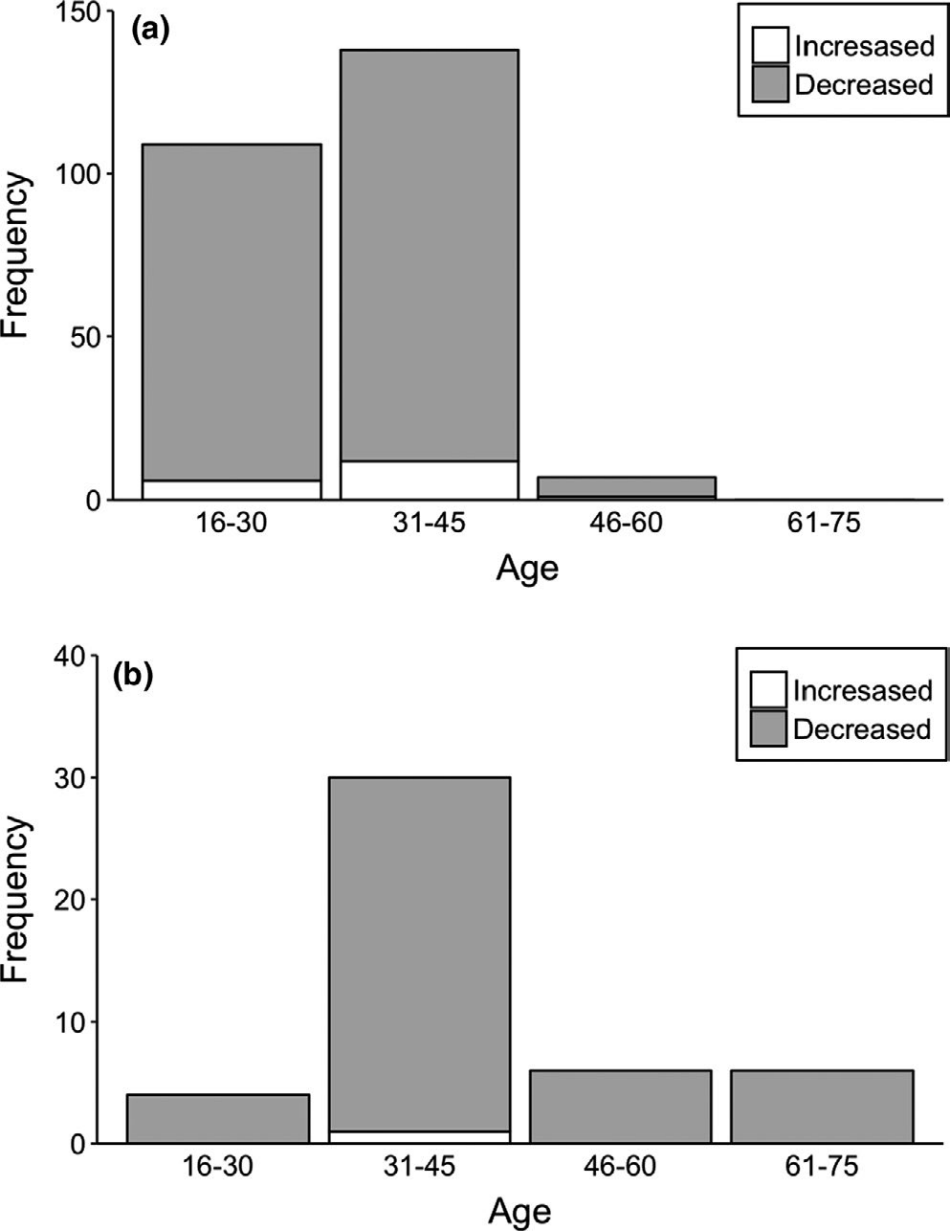
Respondents view whether vulture population in the wild in increasing or decreasing in the study area, (a) formally educated respondents and (b) non‐formally educated respondents

We interviewed to 300 respondents living near to study sites. In the interview, we did not find differences in age between male and female respondents (Kruskal–Wallis test = 1.269, *df* = 1, *p* = .26); however, there was variation in the age according to education (Kruskal–Wallis test = 47.962, *df* = 1, *p* = .001) in respondents (Table 2). In addition, there were differences in formally educated respondents between gender (Fisher’s exact test, *two*‐*tailed* = 0.015) (Table 2). Almost all respondents (>95%) identified the vulture’s pictures during this questionnaire survey. Ninety‐eight percent of respondents reported seeing wild vultures. Among these, formally educated respondents have reported seeing vultures slightly more than nonformally educated respondents (Table 2, Figure 3a,b). Only seven percent of respondents, among which 86% were formally educated, had seen a vulture nest in the wild (Figure 4a,b). Almost all formally educated and noneducated respondents (98%) confirmed the usefulness of vultures in an ecosystem services (Figure 5a,b). Among them, 86% of respondents recognized vultures as important carcass removal agents for environment cleaning, followed by 12% as preventing spread of disease, and two percent not sure. Only three percent of respondents were aware of the negative effects of diclofenac use on vulture populations (Figure 6a,b). Ninety‐six percent of respondents believe vulture populations are declining in Pokhara, Kaski (Figure 7a,b). The respondents suspected the decline was due to habitat loss (58%), toxic food use (20%), others such as forest fire and nestling killing (2%), poisoning (0.6%), and food scarcity (20%). Most of the respondents (84.6%) confirmed the immediate burial of their dead livestock.

**TABLE 2:** Respondents’ knowledge on the Vulture species in Kaski District

Variables	Comparison	Statistics
Age	Male: median = 32; female: median = 33	Kruskal–Wallis test = 1.269, *df* = 1, *p* = .26
Age	Educated: median = 31; nonformally educated: median = 44	Kruskal–Wallis test = 47.962, *df* = 1, *p* = .001
Gender	Educated and nonformally educated	*Fisher’s exact test*, *two*‐*tailed* = 0.015
Vulture seen in the wild	Educated and nonformally educated	*Fisher’s exact test*, *two*‐*tailed* = 0.012

## 4 DISCUSSION

This study confirmed occurrence of eight vulture species in Pokhara Valley and demonstrated that residents are knowledgeable and appreciative of vultures. Among the vulture species, White‐rumped vulture, Egyptian vulture, and Himalayan vulture are critically endangered species and abundantly distributed in areas such as the landfill, nearby Dovilla, and vulture feeding sites (Ghachowk), which likely provide abundant food for them. Populations of most vulture species seem to be increasing since diclofenac was banned in 2006 (Bhusal et al., 2019; Galligan et al., 2019) in Nepal. We did not observe Long‐billed vultures during the study period, probably due to their vagrant nature. This species has been reported in Kaski in 2014 and December 2015 (Dhakal et al., 2019; Inskipp et al., 2020). The Nepal population of Slender‐billed vultures has declined sharply since 1991; previously, the species was a common and widespread resident in Nepal (DNPWC, 2015; Grimmett et al., 2016; Inskipp et al., 2016). Gautam and Baral (2013) indicated the population of Slender‐billed vulture and white‐rumped vulture declined by 53% and 58% respectively between 2006 and 2012 in Pokhara Valley, and in our study, local people also assumed a population decline of all vultures including these species. However, after 2012, the population of Slender‐billed vulture in Kaski District shows a good indication of an increasing population probably due to improved breeding success (Bhusal et al., 2019). We found one nest with a successfully reared nestling in 2017. However, population trends for long‐lived species such as raptors are most sensitive to changes in adult survival (Clark & Martin, 2007; McClure et al., 2017; Sæther & Bakke, 2000). These areas seem a good habitat for vulture occurrence, potentially due to food accessibility near to landfill sites, rivers, and vulture safe feeding site. However, the area is suffering from habitat loss and unsure about the illegal use of Diclofenac. In addition, vultures potentially threats from high collision risk with aircrafts. Pokhara Regional International Airport will start soon its fight from 2022, and vulture movements, nesting, and soaring sites are located within 2 km periphery of Airport. Every year >300 individuals of vultures were recorded in and around Airport site at International Vulture Awareness Day (M. Ghimire, President, Pokhara Bird Society, Pokhara, Personal Communication). To minimize these potential threats on vultures, people’s knowledge and perception is essential.

Knowledge and perception of a species often leads to support for conservation (Bhattarai & Fischer, 2014; Katuwal et al., 2021; Sharma et al., 2019). Therefore, if people understand the usefulness of vultures as an important scavenger (Ballejo et al., 2019; García‐Alfonso et al., 2019), they might take initiation for conservation. In our study, almost all respondents appreciated the usefulness of vultures due to their role in ecosystem services. This knowledge likely stemmed from formal education and from living in a tourist‐influenced area. Indeed, compared to other areas of Nepal, there are more tourists visiting the Pokhara Valley who are interested in vulture sightings. Formally educated people knew more about the vultures. However, sighting of vulture’s nest locations is low probably due to difficulties for nest sighting in tall trees and remote cliffs or actually not breeding near the human‐dominated forest or area. Additionally, more bird conservation activities have been performed in recent years by the Government of Nepal and Bird Conservation Nepal (DNPWC, 2015). In our study too, almost all respondents expected that habitat loss was the major threat for the vulture population decline in Pokhara Valley. Urbanization is one of the major causes of natural habitat loss for wildlife (Gutiérrez et al., 2020; Richards & Belcher, 2020), and it is causing tall and larger trees to be felled around Pokhara Valley.

Burying of deceased livestock helps prevent vultures from feeding on poisoned carcasses but also lessens the amount of food available. Food scarcity is thus another factor for vulture population declines because most of the livestock in recent years have been buried immediately after death. Generally, animal husbandry practices and carcass availability determine vulture distributions (Olea & Mateo‐Tomás, 2009); however, there is a decreasing trend of cattle husbandry in our study area (CBS, 2011). Further, nearly 92% of carcasses are disposed by burying immediately after death in Nepal (Dhakal et al., 2013), and almost 85% of respondents in our survey buried their carcasses. This lack of food across the landscape highlights the importance of vulture restaurants. We only know of one vulture restaurant in Pokhara Valley; however, few people are aware of it, which creates a problem for its sustainability. Therefore, more awareness programs for cattle owners are needed to make the vulture restaurant sustainable.

The richness and abundance of vulture species within the Pokhara Valley of Kaski District highlight its global importance to populations of these birds. Given the awareness and regard for vultures we observed, the Pokhara Valley will likely support long‐term conservation of vulture populations in Kaski District. However, differing levels of knowledge between formally and nonformally educated people suggest more awareness programs are needed, especially for practitioners of animal husbandry who might provide livestock to the vulture restaurant.

## CONFLICT OF INTEREST

None.

## AUTHOR CONTRIBUTIONS

**Hemanta Dhakal:** Conceptualization (equal); data curation (lead); funding acquisition (equal); investigation (lead); writing – original draft (equal); writing – review and editing (equal). **Hari Prasad Sharma:** Conceptualization (equal); formal analysis (equal); supervision (lead); writing – original draft (equal); writing – review and editing (equal). **Christopher J. W. McClure:** Data curation (equal); formal analysis (equal); writing – review and editing (equal). **Munir Virani:** Writing – review and editing (equal). **Brian W. Rolek:** Data curation (equal); formal analysis (equal); software (lead); writing – review and editing (equal). **Narendra Man Babu Pradhan:** Writing – review and editing (equal). **Krishna Prasad Bhusal:** Writing – review and editing (equal).

## Supporting information

Supplementary Material

## Data Availability

A full description of the model and script for model implementation are archived at https://github.com/The‐Peregrine‐Fund/Nepal‐Vultures.
