# Supplementary material for: Vulture distribution and people perception of vultures in Pokhara Valley, Nepal
Source: Ecol Evol. 2022 Jan 12;12(1):e8528. doi: 10.1002/ece3.8528 (PMC8809430; doi:10.1002/ece3.8528)
Supplement: Supplementary file 1 — Supplementary Material [file ECE3-12-e8528-s001.docx]

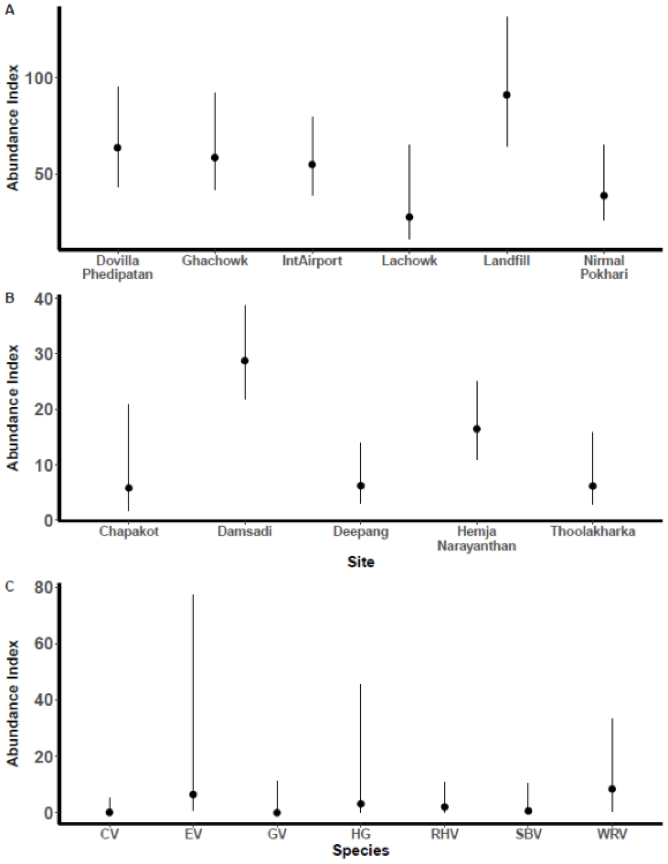


Fig. S1. Panels A and B depict the overall relative abundances of all vultures per survey site. These relative abundances represent the median value of a given species during individual surveys. Thin lines represent the range between the 2.5^th^ and 97.5^th^ percentiles. Note that the axes in panels A and B are scaled differently such that sites with higher vulture abundances are in panel A. Panel C depicts the relative abundances for each species across all sites. CV = Cinareous Vulture, EV = Egyptian Vulture, GV = Griffon Vulture, HG = Himalayan Vulture, RHV = Red-headed Vulture, SBV = Slender-billed Vulture, and WRV = White-rumped Vulture.

Table S1. Median, 2.5^th^, and 97.5^th^ percentiles of overall relative abundance of all vulture species per survey site within the Kaski District, Nepal.

| Site | 2.5^th^ | Median | 97.5^th^ |
| --- | --- | --- | --- |
| Landfill | 67.78 | 93.34 | 129.97 |
| Dovilla Phedipatan | 43.25 | 61.59 | 89.79 |
| Ghachowk | 43.29 | 57.85 | 79.99 |
| IntAirport | 40.60 | 55.66 | 76.60 |
| Nirmal Pokhari | 27.50 | 38.74 | 56.37 |
| Damsadi | 23.71 | 30.31 | 38.76 |
| Lachowk | 17.15 | 26.94 | 47.17 |
| Hemja Narayanthan | 11.32 | 16.24 | 23.54 |
| Thoolakharka | 3.25 | 6.38 | 14.12 |
| Deepang | 3.30 | 6.31 | 12.52 |
| Chapakot | 1.73 | 5.54 | 18.99 |

Table S2. Median, 2.5^th^, and 97.5^th^ percentiles of the detection parameter $B$ for vulture species across survey sites within the Kaski District, Nepal. BV = Bearded Vulture, CV = Cinareous Vulture, EV = Egyptian Vulture, GV = Griffon Vulture, HG = Himalayan Vulture, LBV = Long-billed Vulture, RHV = Red-headed Vulture, SBV = Slender-billed Vulture, and WRV = White-rumped Vulture.

| Species | 2.5 | Median | 97.5 |
| --- | --- | --- | --- |
| CV | -0.55 | -0.04 | 0.44 |
| EV | -0.05 | 0.15 | 0.35 |
| GV | -1.00 | -0.22 | 0.39 |
| HG | -0.47 | -0.08 | 0.28 |
| RHV | -0.10 | 0.09 | 0.28 |
| SBV | -0.02 | 0.24 | 0.53 |
| WRV | -0.15 | 0.03 | 0.21 |

Table S3. The probability that the ratio of observation to process error was > 10. BV = Bearded Vulture, CV = Cinareous Vulture, EV = Egyptian Vulture, GV = Griffon Vulture, HG = Himalayan Vulture, LBV = Long-billed Vulture, RHV = Red-headed Vulture, SBV = Slender-billed Vulture, and WRV = White-rumped Vulture.

| Species | Probability |
| --- | --- |
| CV | 0.12 |
| EV | 0.21 |
| GV | 0.00 |
| HG | 0.10 |
| RHV | 0.04 |
| SBV | 0.16 |
| WRV | 0.14 |

Appendix S1

Questionnaire used to survey 300 households from 11 different sites (Fig. 1) in September 2017 to August 2018, to investigate the people’s knowledge on Slender-billed vulture conservation.

Section I Demographic condition of the respondents

1. Gender: Male/Female
2. Age
3. Education: Formally educated (At least attempted school for education)/Not-formally educated (did not attempted school)

Section II Respondents knowledge on vulture

1. Could you recognize these species? (Which bird is that? By showing multiple pictures)
2. Did you see the nest of vultures within last five years? (This question was asked after their correct identification on vulture)
3. Are live vultures useful in an ecosystem? If yes, why?
4. Do you notice the increasing /decreasing of vultures in the last 10 years in and around your areas? If yes what are the causes? (Use diclofenac on their livestock and toxic chemicals on carcasses, habitat loss, toxic food use, forest fire and killing, poisoning and food scarcity)

Do you know about the use of Meloxicam? Or Diclofenac?

on livestock and its effect on vulture?

1. What do you do if your livestock died?
